# Bioaccumulation of different congeners of Poly-Brominated Diphenyl Ethers (PBDEs) in muscle tissue of males and females of *Clupea harengus* from the North Sea

**DOI:** 10.1007/s11356-021-14347-x

**Published:** 2021-05-18

**Authors:** Andrea Gaion, Ellana Morgan, Stuart Collier, Davide Sartori

**Affiliations:** 1 University Centre South Devon, Long Road, Paignton, TQ4 7EJ UK; 2 Istituto Superiore per la Protezione e la Ricerca Ambientale–ISPRA, via del Cedro 38, 57122 Livorno, Italy

**Keywords:** PBDE congeners, Fish, Bioaccumulation

## Abstract

In the last decades, high concentrations of flame retardants (PBDE) in marine organisms have caused increasing concern among scientists with regards to their biomagnification potential and to human health. Poly-Brominated Diphenyl Ethers have been widely used in the past as flame retardants in various industrial sectors, and their presence in the biota has been associated with different toxicological effects. In this study, concentrations of 9 congeners of PBDE (183, 85, 153, 154, 99, 100, 47, 66 and 28) and morphometric parameters (Total Length, TL; Fork Length, FL in cm and weight) have been measured in muscle of males and females of Atlantic herring (*Clupea harengus*). Results showed no statistical differences between the concentrations of most of the congeners analysed between the two sexes, except for PBDE 153 (Mean ± standard error in males = 0.034 ± 0.005 μg/kg and in females = 0.086 ± 0.040 μg/kg; p = 0.003). This research contributes to better comprehend the ecotoxicological properties of these molecules and their implications for human consumption.

## Introduction

The ubiquitous application of plastics in any sector of the global economy has resulted in a ceaseless research into those chemicals used to refine the physical properties of products to meet the requirements of the market. These plastic additives are often grouped in wide categories that summarise and characterise the main function of the group. Examples of these additives include stabilisers, aimed to reduce the degradation of polymers susceptible to environmental action (Sigmaaldrich 2020), or plasticisers, used to make plastics softer and more flexible (BPF 2020). Flame retardants are a specific class of additives that have caused concern among environmental scientists since production began in the 1970s. Based on their different functional compounds, there are three main categories of chemical flame retardants: halogenated, organophosphorus and inorganic products; within the halogenated hydrocarbons, the main Brominated Flame Retardants (BFRs) are polybrominated diphenyl ethers (PBDEs) (EFSA 2011).

Due to their persistency in the environment and demonstrated toxicity, most formulations have been banned or regulated: the EU restricted the sale of two commercial mixtures of PBDEs, PentaBDE and OctaBDE, in concentrations higher than 0.1% by mass in 2003, and from 2006, all electrical equipment cannot contain PBDEs in any concentration (EFSA 2020). Despite such control measures, these chemicals are classified as Persistent Organic Pollutants (POPs) and, as such, can still be found in every environmental matrix and in different animal taxa to the present day. The Octanol-water partition coefficient (log K_ow_) for these compounds varies between 6.27 and 6.97 depending on the congener (EPA 2017), and as a consequence of their hydrophobic nature, they showed positive correlation with lipid content, especially in fish. With regard to their bioaccumulation in the marine trophic chain, it has been demonstrated that these chemicals do not tend to accumulate into higher trophic levels when normalised by lipid content (Burd et al. 2019). The detoxification process of these compounds varies according to the species studied and the congener: in fish liver cells, biotransformation PBDE 15 was mediated by the action of the cytochrome P450 enzymes and resulted in the formation of bromophenol and two monohydroxylated dibromodiphenyl ether metabolites, whereas PBDE 47 remained not affected by the cell metabolism (Shen et al. 2012). Exposure of human liver cells in vitro caused the formation of 2,4,5-tribromo phenol, two monohydroxylated pentabrominated diphenyl ether metabolites, and a yet unidentified tetrabrominated metabolite whereas exposure to PBDE 209 did not cause the formation of any metabolites (Stapleton et al. 2009). They are defined as Endocrine Disruptors (ED), and different PBDE congeners have been demonstrated to affect thyroid and hepatic functions, as well as nervous, endocrine and reproductive systems in fish. Embryos of zebrafish (*Danio rerio*) exposed to PBDE 47 showed delayed hatching and reduced growth post-hatching; at 96 h post-fertilisation, larvae exhibited tachycardia, progressing into atrioventricular block arrhythmias, and the flow of cerebrospinal fluid in the neural tube and brain was slower than the control group (Lema et al. 2007). Exposure of *Sparus aurata* fibroblast cell line to PBDE 47 and PBDE 99 produced cytotoxicity, reactive oxygen species (ROS) and the expression molecular markers related to cell cycle (Ruiz et al. 2019).

Like many other marine species, herrings (*Clupea harengus*) can be considered as a bioindicator of environmental quality, as model organism for biological/toxicological studies, and as a possible diet-based source of contaminants for humans. Many studies have demonstrated the correlation between lipid content and organic contaminants in this species, highlighting its important contribution for the transfer of contaminants in the marine trophic chain as well as for humans (Lundstedt-Enkel et al. 2010; Miller et al. 2013). Therefore, it is essential to identify the factors involved in the bioaccumulation and detoxification patterns of PBDEs in this species to better understand the risks for both environmental quality and human consumption. In the present study, concentration of different PBDE congeners have been measured in fillets of commercially available Atlantic herrings (*C. harengus*) originating from the North Sea, to investigate the gender-based factors at the base of their differential proportions in the muscle tissue.

## Methods

### Specimens and morpho-biological parameters determination

Specimens of *C. harengus* (n=38) were purchased from a local supermarket with specific request for animals originating from the same fishing batch. Each animal was processed and Total Length (TL, cm), Fork Length (FL, cm), Measured Weight (MW, g) and gender were determined. Measured weight has been compared with the Theoretical Weight (TW, g) calculated for each specimen with the following formula: TW = a × LT^b, where, LT is the total length, factor a (intercept = 0.0048) and b (slope = 3.1984) (Silva et al. 2013). Subsequently, a portion of muscle tissue was weighted and oven-dried at 60 °C for a minimum of 48 h, until constant weight was reached (Bessey and Vanderklift 2013). Lipid content was determined according to Tölgyessy and Miháliková (2016), where 5 g of fish tissue homogenate was processed with 5 mL of acetone/ethyl acetate mixture (6:4, v/v) and, after addition of inorganic salts (2 g MgSO_4_ and 0.5 g NaCl), the organic phase was separated by centrifugation. An aliquot of the organic phase was dried and the lipid content of the fillet homogenate was determined on a wet weight basis. Percentage of protein content was estimated with the remaining percentage after water and lipid analysis.

### Chemical analysis

Total concentrations of PBDE and 9 congeners (183, 85, 153, 154, 99, 100, 47, 66 and 28) were measured in muscle of males and females of Atlantic herring (*Clupea harengus*) by an accredited laboratory (EN ISO 17025; EN ISO 14001) through solvent extraction (acetonitrile) using a modified QuEChERS technique (Romanelli et al. 2017), followed by analysis by GC-QQQ after purification. In this method, a pre-prepared sample is extracted in acetonitrile. For each sample, 5g of muscle tissue has been processed and analysed; PBDE internal standards were added at the beginning of the extraction and underwent through the whole analytical process. Following the Method 1614A USEPA (2010), the procedure was considered valid for percentage of internal standard recovery between 50% and 150%. The extracts are put through a silica clean-up to remove fats and other interfering compounds. The extract is analysed using GC-QQQ. The Mass Spectrometer is operated in electron impact ionisation (EI) MS/MS mode using Multiple Reaction Monitoring (MRM). Internal and external quality control processes have been followed by the laboratory, according to the accreditation requirements. As part of QA/QC requirements, the method reporting limits, defined as the value at which a concentration is detected, quantified and reported with sufficient statistical accuracy (van Buuren 2017), are the following: PBDE 100 = 0.008 μg/kg, PBDE 153 = 0.02 μg/kg, PBDE 154 = 0.01 μg/kg, PBDE 183 = 0.008 μg/kg, PBDE 28 = 0.006 μg/kg, PBDE 47 = 0.02 μg/kg, PBDE 66 = 0.009 μg/kg, PBDE 85 = 0.009 μg/kg, PBDE 99 = 0.02 μg/kg.

### Statistical analysis

Parametric analysis (T test, 1-Way Anova, Pearson correlation) was conducted after checking assumptions through exploration of data: normality was confirmed with a Shapiro-Wilk test (p>0.05 for normal distribution; Das and Imon, 2013); where present, extreme outliers were removed from the dataset (Cosineau and Chartier 2010), Skewness and Kurtosis (SK) of groups corresponding to a p value <0.05 in the Shapiro-Wilk test were used to evaluate the viability of parametric analysis (-1.96 < SK < +1.96 for parametric analysis; George and Mallery 2010). Where these assumptions were not met, alternative non-parametric tests have been conducted (Mann Whitney U test, Kruskal-Wallis, Spearman correlation). Homogeneity of variances was assessed with a Levene’s test (Gastwirth et al. 2009) for the correct determination of post hoc analysis (Tukey test for variances homogeneous, Dunnett’s test otherwise). An initial Principal Component Analysis (total, males and females) was conducted to estimate the factors and their relations that contributed the most to the variability of the data. Due to insufficient percentage of variance explained (<60 %; Hair et al. 2010), a subsequent bivariate correlation analysis including exclusively PBDEs analysed was conducted individually for males and females. Statistical analysis was conducted using the software SPSS v.26.

## Results

All the results from the morphological and chemical analyses are reported in Table 1.

**Table 1:** Morphological, biological and chemical results obtained from the analysis of *C. harengus*

Sample	Sex	% Water	% Lipid	Fork length (cm)	Total length (cm)	Measured weight (g)	Theoretical. weight (g)	∆ weight (g)	PBDE total (ng/g)	PBDE 183 (ng/g)	PBDE 85 (ng/g)	PBDE 153 (ng/g)	PBDE 154 (ng/g)	PBDE 99 (ng/g)	PBDE 100 (ng/g)	PBDE 47 (ng/g)	PBDE 66 (ng/g)	PBDE 28 (ng/g)
1	M	61.2	17.7	22.6	24.9	158.32	140.90	12.36	0.671	0.079	0.029	0.07	0.083	0.084	0.083	0.228	0.009	0.006
2	F	59.2	14.4	24.7	27.3	224.47	187.19	19.91	1.028	0.077	0.026	0.07	0.105	0.184	0.128	0.383	0.043	0.012
3	F	57.4	8.7	25.9	28.6	260.51	217.85	19.58	7.949	0.858	0.951	0.846	0.964	0.999	0.876	0.988	0.712	0.755
4	M	62.7	13.0	25.1	27.7	208.87	197.06	5.99	1.927	0.084	0.09	0.098	0.172	0.157	0.36	0.812	0.1	0.054
5	F	57.6	13.0	24.4	26.9	228.26	180.02	26.80	0.912	0.05	0.032	0.059	0.099	0.133	0.131	0.382	0.018	0.008
6	F	62.0	11.1	22.6	24.9	170.85	140.90	21.25	0.655	0.03	0.009	0.028	0.057	0.035	0.141	0.34	0.009	0.006
7	M	61.6	13.2	24.9	27.5	207.77	192.08	8.17	1.438	0.042	0.01	0.039	0.094	0.103	0.315	0.816	0.01	0.009
8	F	62.6	15.9	23.5	25.9	190.3	159.64	19.21	0.499	0.073	0.014	0.046	0.052	0.048	0.064	0.187	0.009	0.006
9	F	61.6	11.0	23.6	26.0	193.7	161.82	19.70	0.876	0.057	0.024	0.058	0.098	0.064	0.132	0.428	0.009	0.006
10	M	61.9	18.9	22	24.3	152.74	129.29	18.14	0.43	0.06	0.009	0.043	0.049	0.045	0.046	0.163	0.009	0.006
11	F	62.4	7.4	22.7	25.0	172.21	142.90	20.51	0.465	0.03	0.009	0.02	0.033	0.035	0.086	0.237	0.009	0.006
12	M	58.5	17.2	24.3	26.8	192.98	177.67	8.62	1.469	0.02	0.009	0.032	0.101	0.12	0.272	0.861	0.02	0.034
13	M	56.3	12.0	22.3	24.6	194.39	135.01	43.98	0.896	0.019	0.009	0.026	0.049	0.138	0.161	0.476	0.009	0.009
14	F	60.3	15.5	22.7	25.0	166.77	142.90	16.70	0.485	0.02	0.009	0.021	0.031	0.034	0.106	0.249	0.009	0.006
15	F	60.2	11.2	22.6	24.9	175.2	140.90	24.34	0.526	0.012	0.009	0.02	0.046	0.066	0.087	0.248	0.017	0.021
16	F	58.5	15.0	25	27.6	245.17	194.56	26.01	0.635	0.02	0.009	0.02	0.05	0.053	0.129	0.319	0.016	0.019
17	M	61.5	10.9	22.6	24.9	167.83	140.90	19.11	0.584	0.019	0.015	0.023	0.054	0.047	0.133	0.255	0.016	0.022
18	M	60.1	13.8	22.1	24.4	169.05	131.18	28.87	0.609	0.02	0.015	0.027	0.047	0.086	0.094	0.266	0.026	0.028
19	M	60.6	13.2	24.1	26.6	216.6	173.04	25.17	0.58	0.025	0.01	0.022	0.048	0.075	0.078	0.269	0.023	0.03
20	F	61.4	16.0	21.4	23.6	153.5	118.35	29.70	0.468	0.04	0.015	0.031	0.044	0.067	0.071	0.167	0.017	0.016
21	M	60.8	12.4	23	25.4	194.9	149.03	30.78	0.589	0.012	0.009	0.02	0.05	0.04	0.14	0.284	0.014	0.02
22	F	60.6	14.2	23	25.4	189.6	149.03	27.22	1.192	0.02	0.009	0.02	0.067	0.105	0.232	0.673	0.033	0.033
23	F	59.7	10.7	23.5	25.9	212.02	159.64	32.81	0.57	0.03	0.014	0.023	0.052	0.066	0.092	0.249	0.02	0.024
24	M	61.1	19.6	21.9	24.2	180.8	127.42	41.90	0.504	0.02	0.009	0.02	0.039	0.053	0.073	0.246	0.021	0.023
25	F	63.0	20.4	23.8	26.3	174.5	166.25	4.96	0.559	0.012	0.009	0.02	0.046	0.058	0.092	0.28	0.02	0.022
26	M	60.5	11.1	23.7	26.2	198.52	164.02	21.03	0.996	0.026	0.009	0.031	0.06	0.127	0.169	0.491	0.043	0.04
27	M	63.2	10.3	22.6	24.9	175.2	140.90	24.34	0.83	0.017	0.009	0.023	0.065	0.104	0.127	0.411	0.037	0.037
28	M	58.1	28.8	22.5	24.8	205.02	138.92	47.58	0.49	0.02	0.009	0.02	0.046	0.063	0.077	0.215	0.019	0.021
29	F	63.7	8.7	26	28.7	211	220.55	-4.33	2.389	0.008	0.009	0.02	0.099	0.079	0.663	1.45	0.028	0.033
30	F	60.8	5.8	23.3	25.7	208.1	155.34	33.97	2.571	0.292	0.374	0.384	0.442	0.347	0.288	0.317	0.103	0.024
31	F	60.5	9.7	23.2	25.6	205.88	153.21	34.37	1.149	0.03	0.076	0.096	0.094	0.19	0.167	0.387	0.075	0.034
32	F	61.1	13.3	22.1	24.4	177.27	131.18	35.14	0.577	0.01	0.009	0.02	0.04	0.055	0.102	0.3	0.02	0.021
33	M	62.1	9.4	26	28.7	233.39	220.55	5.82	0.884	0.03	0.009	0.03	0.075	0.119	0.108	0.336	0.142	0.035
34	M	60.8	14.8	21.5	23.7	180.66	120.13	50.39	0.775	0.01	0.009	0.02	0.48	0.069	0.106	0.034	0.024	0.023
35	F	61.2	19.0	24.4	26.9	205.81	180.02	14.33	0.406	0.008	0.009	0.023	0.036	0.049	0.062	0.189	0.016	0.014
36	F	62.0	19.5	22.7	25.0	192.34	142.90	34.59	0.376	0.008	0.009	0.023	0.03	0.038	0.048	0.187	0.019	0.014
37	F	62.5	12.9	22.4	24.7	176.23	136.95	28.68	0.551	0.008	0.009	0.023	0.03	0.067	0.084	0.285	0.022	0.023
38	F	61.2	10.8	24.4	26.9	233.981	180.02	29.97	0.481	0.008	0.009	0.023	0.036	0.067	0.06	0.242	0.016	0.02

**Fig. 1:**
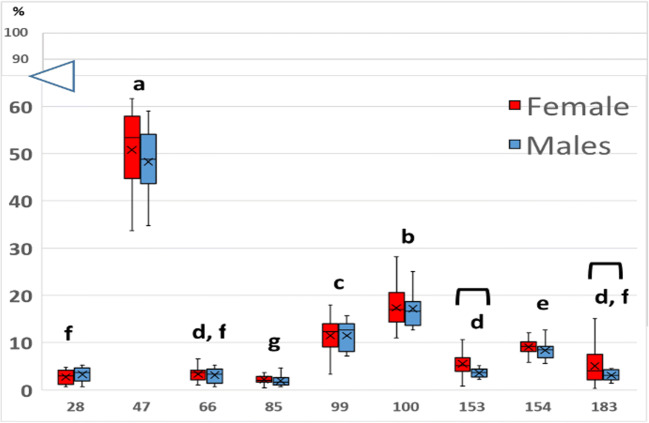
Percentages of different PBDE congeners in *C. harengus.* Significant difference (p<0.05) between sexes is highlighted with brackets, whereas significant differences between percentages of congeners is evidenced with different letters.

Statistical analysis showed that there was no statistical difference (p ≥ 0.05) between males and females in terms of percentage of water (61.19 ± 1.16% and 61.05 ± 1.50% respectively), percentage of lipids (14.77 ± 1.21% and 12.91 ± 0.83%), FL (23.20 ± 0.33 cm and 23.54 ± 0.25 cm), MW (189.82 ± 5.52 g and 198.53 ± 5.91 g), deviation of MW from TW, concentration of PBDE total and congeners; only PBDE 153 resulted to be significantly different between the 2 sexes (Mean ± standard error in males = 0.034 ± 0.005 μg/kg and in females = 0.086 ± 0.040 μg/kg; p = 0.003). Concentrations of congeners were distributed according to the following descending order (p < 0.001): PBDE 47 > PBDE 100 > PBDE 99 > PBDE 154 > PBDE 28-66-153-183 > PBDE 85 (Fig. 1).

A Principal Component Analysis has been attempted to reduce the dimensionality of factors, and the total variance explained by the first two components was 47.95%, 47.1% and 59.78% for all the animals pooled together, males and females respectively. As the total variance explained was lower than acceptable levels of 60% (Hair et al. 2010), a bivariate correlation were produced for all the molecules analysed specifically for males and for females (Table 2).

**Table 2:**
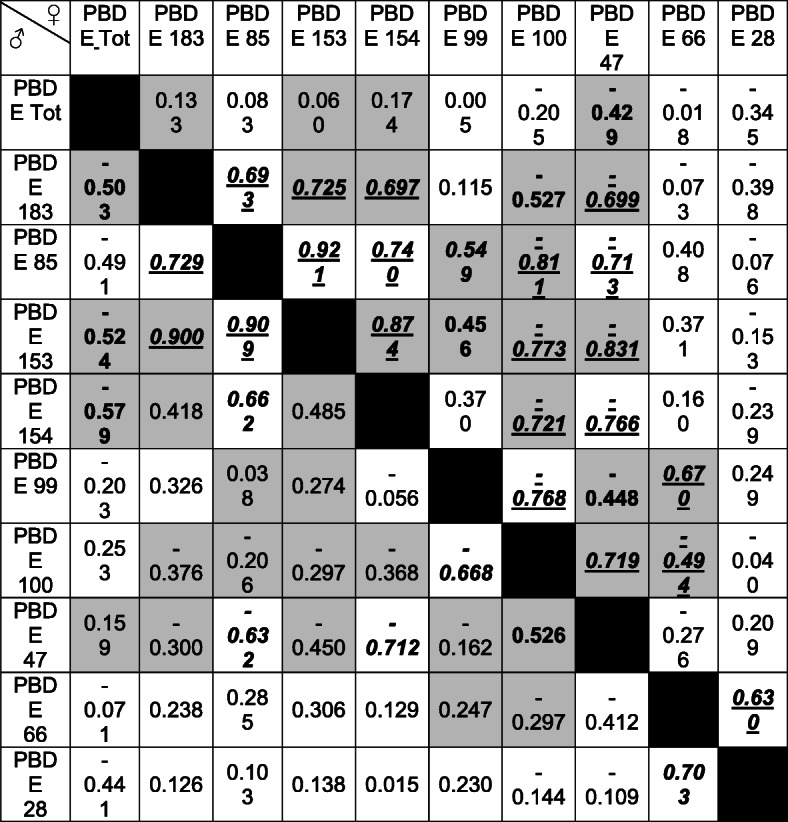
Correlation coefficients in males (♂) and females (♀). Significant values are reported in **bold** (<0.05), in ***bold italics*** (<0.01) and in ***bold italics underlined*** (<0.001). Cells reporting discrepancies between correlation coefficients in males and females have been highlighted with a grey background

This analysis revealed that numerous variables correlated differently in males and females (e.g. Total PBDE and Deviation from TW in females r = -0.016, in males r = -0.552; Total PBDE and PBDE 153 in females r = 0.060, in males r = -0.524).

## Discussion

This study contributed to enhance the knowledge around the biological and physiological parameters that may affect the bioaccumulation of PBDEs in *C. harengus*. As demonstrated by the homogeneity of morphometrical parameters, specimens measured in this study originated from the same fishing event and belonged to the same stock (product information). Overall, the mean percentage of water in the muscle tissue was 61.12 ± 0.22% and percentage of lipid 13.28 ± 0.59%, with no statistical difference between males and females. Lipid content is the most contributing factor which correlates with PBDEs accumulation (Zeng et al. 2013) and, although, the values reported in this study are in line with published data (Murray and Burt 2001; Rajasilta et al. 2018), particular consideration should be taken when interpreting PBDEs concentrations in the light of the high natural variability of lipid content and the analytical method used (Nielsen et al. 2005). It has been demonstrated that interannual variability of lipid could be in the range of 1.9% and 11.7%, with some Authors reporting a range between 1% and 25% (Nielsen et al., 2005; Rajasilta et al. 2018). Therefore, it could be very beneficial to understand how the kinetic of bioaccumulations can vary in response to different seasonal lipid content, measured with a standardised protocols. In addition, seasonal development of gonadal tissue in males or females of *C. harengus* can importantly regulate the percentage of lipids in muscle tissue (Henderson and Almatar 1989).

Total PBDE concentrations for all the specimens in this study (Table 1) exceeded the Environmental Quality Standard (EQS) value of 0.0085 μg/kg reported for biota in the Directive 2013/39/EU of the European Parliament and of the Council for priority substances (Directive 2013/39/EU 2013), with a percentage of excess ranging from 3276.47% to 27241.18%. This reference value is set for the sum of the concentrations of congener numbers 28, 47, 99, 100, 153 and 154 only, not including the congeners 66, 85 and 183 analysed here; however, this inconsistency is irrelevant as, apart from the congener 28 in 8 animals, the concentration of each single congener in all the samples exceeded the EQS single-handily. The fish analysed in this study originated from the North Sea and the average total PBDEs concentration was 1.00 μg/kg (0.97 μg/kg considering only the congeners regulated by the European directive); these values are slightly lower than the concentrations referring to animals originating from the southern Baltic Sea (1.2 μg/kg; Szlinder-Richert et al. 2010). Although concerning, these values are similar or lower to other published data: in 2011 the European Food Safety Authority conducted an investigation about PBDE in foods, and found values of 1.03 μg/kg for the conger 47 in herrings, compared to a mean value of 0.39 μg/kg reported in this study (EFSA 2011). In the same study, the authors conducted a thorough analysis of the toxicological and epidemiological properties of flame retardants for animals and humans, identifying the liver, thyroid hormone homeostasis, and the reproductive and nervous system as the main targets for PBDE toxicity and indicating that, although these contaminants do not induce gene mutations, they can result in DNA damage through the induction of reactive oxygen species (ROS).

In accordance with the reported studies, the most accumulated congener in this study was PBDE 47, with no statistical difference between males and females. This compound has proven to be particularly toxic for correct larval development of fish species, with the hydroxylated BDE-47 compound, namely 6-OH BDE-47, able to affect neurodevelopment of larvae of zebrafish (Mhadhbi et al. 2012; Yang et al. 2017). On the contrary, statistical difference between the two sexes was measured for the percentages of the congeners 153 and 183, with females showing higher percentages compared to males (p < 0.05). Assuming that males and females originated from the same stock, there can be two possible explanations for this result. The first one is that the two sexes feed on diverse preys, possibly originating from separate areas, characterised by different levels of these two congeners. Indeed, it has been demonstrated that PBDE 183 could be an indicator congener of octa mixtures, which are primary used in the electronic industry (Kwan et al. 2014). In another study, the authors associated a high presence of the congener PBDE 153 in juveniles and adult males of loggerhead turtle that foraged between Carolina and New Jersey (USA), as a consequence the presence of this molecule could also be a distinctive indicator in organisms feeding on a specific area (Stewart et al. 2011). In addition, in an experiment conducted on rats, the latter was demonstrated to be one of the congeners undergoing the least metabolism (Sanders et al. 2006). This possibility could be corroborated by the statistical difference in concentration of PBDE 153 measured between males and females (Mean ± standard error in males = 0.034 ± 0.005 μg/kg and in females = 0.086 ± 0.040 μg/kg; p = 0.003). Alternatively, the gender-based disparity could reflect different metabolic pathways for males and females, representing the first step towards a more comprehensive understanding of the toxicological risk for marine organisms associated with PBDE. Toxicokinetic studies of these molecules are lacking in fish, but, based on evidence from rodents, it has been demonstrated that PBDE 153 accumulated more than congeners 47, 99, 100 mainly due to differential excretion between congeners (Staskal et al. 2006). In herrings, storage lipids sustain routine and active metabolism, and this can vary with gender and different maturation stages (Bradford 1993) and, as PBDEs are importantly correlated with lipid content, their bioaccumulation and toxicokinetic can be altered by different metabolic pathways. The hypothesis of profound differences in metabolism between males and females of *C. harengus* is additionally supported by the numerous discrepancies among correlation coefficients between different molecules (Table 2). For example, total PBDE correlates significantly with PBDE 153 and 183 exclusively in males (r = -0.524 and r = -0.503 respectively), whereas correlations between congener 47 and PBDE 99, 153 and 183 were only significant in females (r = -0.448, r = -0.831 and r = -0.699 respectively). Our aim is to consider this preliminary study as a base to further investigate this aspect by expanding the analytical approach with stable isotope analysis of the two genders, analysis of gonadal developmental stages and age determination with otolith reading. An additional development of this analytical approach could be the measurement of different chemical species (various oxidation states or functional groups) of congeners to be used as finger-print for individual compound bioaccumulation. Further studies aimed to enhance the understanding of the differences in metabolic pathways as well as different toxicokinetic of flame retardants between males and females of *C. harengus* are needed to better comprehend the ecotoxicological properties of such omnipresent persistent organic contaminants and their implications for human consumption.

## Data Availability

The datasets used and/or analysed during the current study are available from the corresponding author on reasonable request.
